# Self-administered acupressure for chronic severe functional constipation: A study protocol for a randomized controlled trial

**DOI:** 10.1097/MD.0000000000026349

**Published:** 2021-06-25

**Authors:** Weifeng Hu, Xiaoming Ying, Jialei Sun, Binghua Fan, Rubao Guo

**Affiliations:** a The Third School of Clinical Medicine (School of Rehabilitation Medicine), Zhejiang Chinese Medical University; b Department of Tuina; c Department of Intervertebral Discs, The Third Affiliated Hospital of Zhejiang Chinese Medical University, Hangzhou City, Zhejiang Province, China.

**Keywords:** acupressure, functional constipation, protocol, randomized controlled trial

## Abstract

**Background:**

Functional constipation is a disease with a high incidence, which has a bad effect on general health, mental health, and social functioning. However, current treatment is sometimes unsatisfactory. Acupuncture has been proven effective in some randomized controlled trials. Acupressure is a subtype of acupuncture and can be manipulated by the patients at home. But the evidence is limited now. This study aims to provide some strict evidence for the use of self-administered acupressure in the treatment of functional constipation.

**Methods:**

This 2-armed, parallel, nonspecific controlled, randomized trial will be conducted at The Third Affiliated Hospital of Zhejiang Chinese Medical University in Hangzhou. A total of 154 FC patients will be enrolled into the acupoint group and the sham acupoint group with a ratio of 1:1 into this trial and it will consist of a 2-week run-in period, an 8-week intervention period, and an 8-week follow-up period. The treatment will be done by the patients themselves at home twice a day and they should sign in on the WeChat APP every day to make sure they have done the acupressure. The outcome will also be collected in WeChat APP through the diary and questionnaires. For the one who is unable to use the WeChat, the print edition of the diary and questionnaires are provided and the supervision will be done by the short message. The primary outcome will be the proportion of participants whose CSBM≥3 during week 3 to 10. The secondary outcome will be the proportion of participants whose CSBM ≥3 between 2 groups in week 11 to 18, Spontaneous bowel movements, Bristol Stool Form Scale, Straining severity scores, Patient assessment of constipation quality of life, and Medicine use.

**Discussion:**

Acupressure is not an invasive method and can be done by the patient itself at home. We hope this trial will provide credible evidence to the application of self-acupressure for the management of severe chronic functional constipation.

**Trial registration:**

This trial has been registered at the Chinese Clinical Trial Registry (ChiCTR2000038594).

## 1 Introduction

Functional constipation (FC) is a disease without an organic etiology and is accompanied by infrequent bowel movements, hard stools, and difficult defecation. It is now believed to be caused by a disorder of gut-brain interaction and the diagnosis of FC, which should rule out irritable bowel syndrome with constipation, opioid-induced constipation, and functional defecation disorders.^[1]^ Severe chronic FC is defined as fewer than 3 complete spontaneous bowel movements (CBSM) per week for more than 3 months.^[2]^ According to a large meta-analysis of 45 population-based surveys, almost 14% of the global population is affected by chronic constipation.^[3]^ There are 1 million visits for chronic constipation in the United States annually.^[4]^ This disease involves large medical costs and has a negative effect on general health, mental health, and social functioning.^[5]^ Moreover, constipation may also increase the risk of cardiovascular diseases, such as venous thromboembolism.^[6]^

Current management of chronic constipation includes lifestyle modification (fiber intake, fluids, and exercise), pharmacology therapies (bulking agents, osmotic laxatives, stimulant laxatives, prokinetics, and secretagogues), biofeedback therapy, and surgery.^[7]^ However, medication for chronic constipation sometimes cannot satisfy patients due to inadequate or temporary relief of the symptoms.^[7]^ Moreover, medication results in some side effects. Biofeedback therapy is effective for rectal evacuation disorders, but it may require multiple clinic visits and is only available in some specialized centers.^[1]^ As monotherapies have limitations and cannot satisfy all the needs of patients, alternative treatments have become increasingly popular.^[8]^

Acupuncture has long been used in the management of constipation in China. It has been proven effective in some randomized controlled trials (RCTs).^[9,10]^ Acupressure is a subtype of acupuncture. It uses fingers, elbows, and hands to press specific acupoints to open up the flow of qi to treat constipation. It has been proven effective in constipation patients with advanced cancer,^[11]^ as well as in pregnant^[12]^ and psychiatric patients.^[13]^ Compared with acupuncture, acupressure can be performed by oneself at home instead of at specific clinics. However, the number of RCTs remains small, and the trials have a high/unclear risk of bias. Thus, we designed an RCT to investigate the safety and efficacy of self-administered acupressure to improve symptoms of FC. Furthermore, we hope that this trial will provide evidence for the integrated use of self-administered acupressure to treat FC.

## 2 Methods/design

### 2.1 Study design

**Figure 1:**
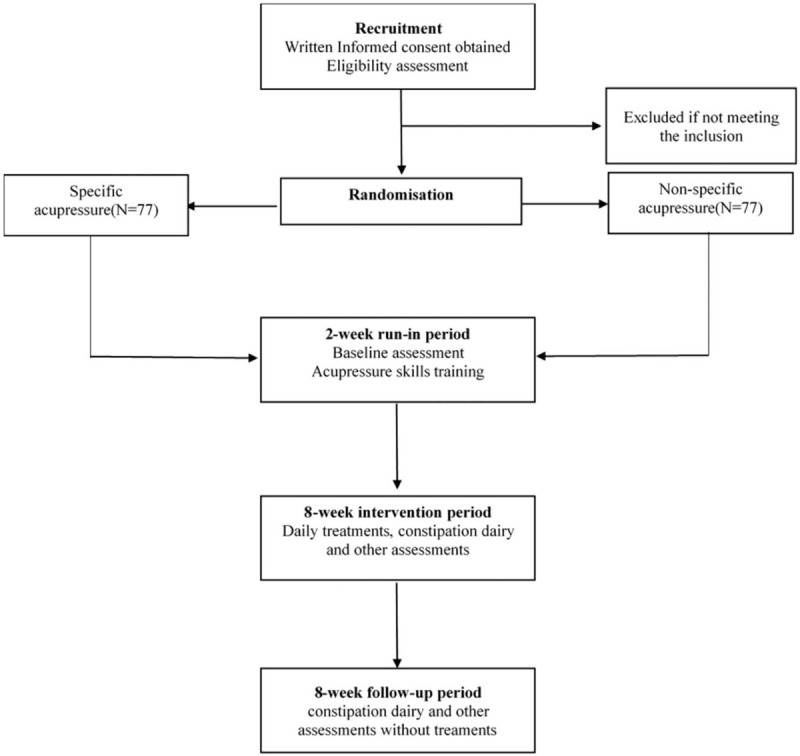
Flowchart of the trial.

This study will be a randomized, 2-arm, parallel, sham-controlled trial. This study will be conducted at The Third Affiliated Hospital of Zhejiang Chinese Medical University from July 2021 to July 2023. A total of 154 FC patients will be enrolled into 2 groups (acupoint group and sham acupoint group) at a ratio of 1:1 in this trial, which will consist of a 2-week run-in period, an 8-week intervention period, and an 8-week follow-up period. The Ethics Committee of the Third Affiliated Hospital of Zhejiang Chinese Medical University approved this study (ZSLL-ZN-2021-002-01). The first edition of the protocol, which was registered at the Chinese Clinical Trial Registry (ChiCTR2000038594) on September 30, 2019, was developed as required by the Standard Protocol Items: SPIRIT 2013. The study design is illustrated in a flow chart in Figure 1.

### 2.2 Participants and recruitment

#### 2.2.1 Primary process of recruitment

Participants will be recruited from the general population of Hangzhou Zhejiang. We will publicize the trial in multimodal strategies, including WeChat official accounts, newspapers, and recruitment posters at outpatient departments or inpatient wards.

The participants who are interested in the trial will contact us via WeChat or mobile phone. Informed consent must be obtained before the first interview. We will check if the participant meets the Rome IV Criteria and select the participant according to the inclusion and exclusion criteria. After the former procedure, the participant will enter the 2-week run-in period, during which the baseline information of the defecation will be collected, and the registered acupuncturist will teach the participants the location of the point and the details of the acupressure manipulation. The baseline assessment includes CBSMs, spontaneous bowel movements, the Bristol Stool Form Scale (BSFS),^[14,15]^ straining severity scores, and patient assessment of constipation quality of life (PAC-QOL).^[16]^ The study schedule is summarized in Table 1.

**Table 1:** Schedule of recruitment, intervention, and assessments.

	Study period																		
TIMEPOINT	Interview	Baseline		Treatment								Follow-up							
Week		1	2	3	4	5	6	7	8	9	10	11	12	13	14	15	16	17	18
RECRUITMENT	×																		
Informed Consent	×																		
Eligibility Screen	×																		
Demography	×																		
Medical History	×																		
Randomization		×																	
INTERVENTIONS																			
Acupoint self-administered acupressure				×	×	×	×	×	×	×	×								
Sham acupoint self-administered acupressure				×	×	×	×	×	×	×	×								
ASSESSMENT																			
The proportion of participant whose CSBM≥3		×	×	×	×	×	×	×	×	×	×	×	×	×	×	×	×	×	×
SBM		×	×	×	×	×	×	×	×	×	×	×	×	×	×	×	×	×	×
BSFS		×	×	×	×	×	×	×	×	×	×	×	×	×	×	×	×	×	×
Straining severity scores		×	×	×	×	×	×	×	×	×	×	×	×	×	×	×	×	×	×
PAC-QOL				×			×				×				×				×
Medicine usage		×	×	×	×	×	×	×	×	×	×	×	×	×	×	×	×	×	×

#### 2.2.2 Diagnostic criteria

The diagnostic criteria will be according to the Rome IV Criteria for FC, which was revised from Rome III by the Rome Foundation in 2016.^[1]^

#### 2.2.3 Inclusion criteria

The inclusion criteria are as follows:

(1) Satisfaction of Rome IV Criteria for FC
(2) Two or fewer mean weekly Rome IV Criteria for more than 3 months
(3) Age between 18 and 65 years
(4) No administration of rescue medicine (glycerol or sorbitol anal enema) 48 hours before the trial or any medication for constipation before enrollment for at least 2 weeks
(5) No administration of any other therapy for constipation, such as acupuncture, acupressure, or biofeedback therapy, and no participation in any other trial in the previous 3 months.
(6) Documented consent to participate in the whole study

#### 2.2.4 Exclusion criteria

The exclusion criteria are as follows:

(1) Constipation possibly caused by irritable bowel syndrome, or inflammatory or structural bowel diseases
(2) Constipation possibly caused by drugs or endocrine, metabolic, neurologic, or postoperative diseases
(3) Presence of tumors, severe cardiovascular, hepatic, renal diseases, or other severe diseases
(4) Inability to cooperate because of cognitive dysfunction, aphasia, mental disorders, or other illness
(5) Pregnant women, lactating women, or those who plan to get pregnant in the following 3 months
(6) Presence of abdominal aortic aneurysm or hepatosplenomegaly
(7) Skin lesions at the operating site of the acupoints

### 2.3 Intervention

After randomization, the registered acupuncturist will teach the participants the location of the acupoints and the details of the acupressure manipulation in the first 2-week run-in period. The participants will be provided with pictures, text descriptions, and videos about the acupressure manipulation for further study at home. At the end of the second week, the participants will be asked to go to the clinical trial laboratory to be checked by the registered acupuncturist to manipulate the acupressure accurately. Self-acupressure will be performed twice a day during the intervention period, and the patient should sign in on WeChat every day. If the participant is unable to use WeChat, the print edition of the diary and questionnaires will be provided, and the supervision will be performed by short messages. Participants who do not have 3 or more bowel movements can use 100 mL glycerol or 40 to 60 mL sorbitol anal enema with detailed documentation.

#### 2.3.1 Details of the acupressure

In addition to the international standard of the location of the acupoints, the proper acupoints will be selected by the sensation of pressing. The proper acupoints should also be more sensitive than the surrounding areas. The thumb or middle finger can be used to perform acupressure. The force of self-pressing must be sufficiently strong but still within a comfortable range. The participants will be instructed to perform acupressure using circular movements. Each acupoint will be pressed for 1 minute.

#### 2.3.2 Experimental group: Acupoint self-administered acupressure group

The 3 acupoints have been selected according to the consensus of acupuncture experts and a previous RCT on acupuncture for chronic constipation.^[17]^ The acupoints are as follows: bilateral Tianshu (ST25), bilateral Fujie (SP14), and bilateral Shangjuxu (ST37).

#### 2.3.3 Control group: Sham acupoint self-administered acupressure group

The acupoints are as follows: bilateral sham Tianshu (ST25), bilateral sham Fujie (SP14), and bilateral sham Shangjuxu (ST37). All the sham points are 1 cun outside parallel to the real point.

#### 2.3.4 Symptomatic relief medications

Participants who do not have 3 or more bowel movements will be allowed to use 100 mL glycerol or 40 to 60 mL sorbitol anal enema.

### 2.4 Outcome

Participants will be required to complete a diary of constipation throughout the trial on WeChat. Most outcomes will be extracted from the diary. Moreover, some questionnaires must be completed. If the participant is unable to use WeChat, the print edition of the diary and questionnaires will be provided.

#### 2.4.1 Primary outcome

The primary outcome of this trial is the proportion of participants whose CSBM is ≥3 during weeks 3 to 10 (bowel movements with a sensation of complete evacuation).^[2]^

#### 2.4.2 Secondary outcome

##### 2.4.2.1 Spontaneous bowel movements

Spontaneous bowel movements refer to bowel movements without using the medication for symptomatic relief of constipation during weeks 3 to 18.

##### 2.4.2.2 CSBM in weeks 11–18

This signifies comparing the proportion of participants with CSBM ≥3 between the 2 groups in weeks 11 to 18.

##### 2.4.2.3 BSFS

The BSFS has 7 pictures of different styles of stools to facilitate the recording of stool consistency. Each picture had its grade. The first type is the one with the hardest stool, and the seventh type is entirely liquid. It will be recorded in grades per week during weeks 3 to 18. The higher the grade, the harder the stool.^[15]^

##### 2.4.2.4 Straining severity scores

The straining severity scores aim to measure the degree of difficulty of defecation during weeks 3 to 18. Straining will be rated with scores of 0, 1, 2, and 3, which indicate “not difficult,” “a little difficult,” “difficult,” and “very difficult,” respectively.

##### 2.4.2.5 PAC-QOL

The PAC-QOL contains 28 items to assess the effect of constipation on 4 subscales: physical discomfort, psychosocial discomfort, worries and concerns, and satisfaction. Each item has a score ranging from 0 to 4. A lower score indicates a better quality of life. The PAC-QOL will be assessed at weeks 2, 6, 10, 14, and 18.

##### 2.4.2.6 Medicine use

The proportion of participants and the weekly frequency of rescue medication use and other defecation measures will be recorded in the diary.

### 2.5 Sample size calculation

In this trial, pass 11 was used to calculate the sample size. There is an absence of studies on acupressure for chronic FC. The selection of the acupoint and the sham acupoint of our study is the same as that of a previous acupuncture study,^[17]^ as is the primary outcome. In this trial, we wanted to achieve 80% power at a significance level of 0.05. Considering a 10% dropout rate, we need 154 subjects in total and 77 subjects per group.

### 2.6 Randomization and blinding

The participants will be enrolled in the experimental and control groups at a ratio of 1:1. An independent researcher will generate the randomization sequence using SPSS (SPSS 21.0, SPSS Inc., Chicago, IL). The random numbers will then be placed in opaque envelopes, which will be numbered sequentially and sent to the independent researcher in charge of randomization. The envelopes will be opened sequentially according to the numbers. Then, allocation is determined. The former researcher will unmask the envelopes after interviewing; therefore, the participants and other investigators will be blinded to the allocation. Outcome assessors, data managers, and statisticians will be blinded in this trial and will be separated so that they cannot share information. Furthermore, we will set a standard procedure for communication to ensure treatment blinding. The allocation will be revealed once the trial is completed.

### 2.7 Safety evaluation

The participants at home will self-administer acupressure. The participants will be told that if any adverse events (AEs) occur, they should communicate with the researchers at once. Regardless of whether the AE is related to the treatment, the treatment should be terminated when AE occurs. The participants should receive appropriate treatment at once. In 24 hours, the researcher should report the AE to ethical committees that decide whether the participants should drop out of the trial. AEs will also be recorded in a case report form.

### 2.8 Date collection and management

Data will be extracted from the diary of constipation and questionnaires. All dates will be recorded in the case report forms (CRFs) stored in the lock-up cabinet. Details of the CRF writing will be formed and taught. At least 2 data administrators will enter the information independently and proofread it. The electronic version of CRFs will be stored in the Third Affiliated Hospital of Zhejiang Chinese Medical University password-protected computer, which is available only to the investigator in charge of data management.

The Department of Science and Education in The Third Affiliated Hospital of Zhejiang Chinese Medical University, which is not participating in the study, will monitor the data, including the CRFs, protocol compliance, data management, treatment administration, and AEs.

The information of the grouping and the results of the study will be provided to the participants after the trial. Publications will only report aggregated data, and personal identities will not be disclosed.

### 2.9 Date analysis

SPSS (version 21.0; SPSS Inc., Chicago, IL) will be used in statistical analyses by an independent statistician. Continuous variables will be presented as mean ± standard deviation, while categorical variables will be represented as percentages and numbers. The Chi-square test will be used to assess categorical variables, such as the characteristic and demographic data. The *t* test or Wilcoxon rank-sum test will be used for within- and inter-group comparisons according to the homogeneity and normality analysis. The date of the participant receiving at least one treatment will be included in the analysis. Missing data will be dealt with using the worst-case scenario method. We set the confidence interval to 95%. All tests are 2-tailed, and *P*-values <.05 will be considered to be significantly different.

## 3 Discussion

Chronic constipation has a negative impact on the quality of life, similar to some organic diseases such as diabetes and depression, and places a heavy medical burden on society.^[18]^ Increasing attention is being paid to alternative treatments (such as massage and acupuncture) for constipation, and new therapies are becoming increasingly popular.^[19]^ Acupuncture has been proven to be noninferior to prucalopride in relieving severe chronic constipation in a recent study.^[20]^ However, acupuncture for constipation can be performed only in specialist clinics, making it inconvenient for the patient. Both acupuncture and acupressure are based on meridian theory in Chinese medicine and influence the flow of qi by stimulating acupoints. However, acupressure is not an invasive method and can be performed by the patient at home. As one group of trials contains the sham acupoints, it is impossible to blind the researchers responsible for the teaching of acupressure. Patients will not know what group they are in. We will also blind data managers and the statistician. They will not communicate the trial's outcome. Moreover, the patient will assess the outcome, which is less likely to introduce biases to subjective outcomes.^[21]^ We will not perform the flora analysis due to the limitation of the fund, although it is an ideal way to explore the mechanisms of acupressure for constipation management.

High-quality data will be collected from this randomized, 2-arm parallel, sham-controlled trial to determine whether acupressure is effective for severe chronic FC. We hope that this trial will provide credible evidence for the application of self-acupressure for the management of severe chronic FC.

## Author contributions

**Conceptualization:** Weifeng Hu, Binghua Fan.

**Investigation:** Xianming Ying, Jialei Sun.

**Methodology:** Weifeng Hu, Xianming Ying, Jialei Sun, Xiaoming Ying.

**Supervision:** Rubao Guo.

**Writing – original draft:** Weifeng Hu.

**Writing – review & editing:** Rubao Guo, Binghua Fan.
